# Endothelial cells are intrinsically defective in xenophagy of *Streptococcus pyogenes*

**DOI:** 10.1371/journal.ppat.1006444

**Published:** 2017-07-06

**Authors:** Shiou-Ling Lu, Tsuyoshi Kawabata, Yi-Lin Cheng, Hiroko Omori, Maho Hamasaki, Tatsuya Kusaba, Ryo Iwamoto, Hirokazu Arimoto, Takeshi Noda, Yee-Shin Lin, Tamotsu Yoshimori

**Affiliations:** 1 Department of Intracellular Membrane Dynamics, Graduate School of Frontier Biosciences, Osaka University, Osaka, Japan; 2 Department of Genetics, Graduate School of Medicine, Osaka University, Osaka, Japan; 3 Department of Microbiology and Immunology, College of Medicine, National Cheng Kung University, Tainan, Taiwan; 4 Department of Biotechnology and Laboratory Science in Medicine, School of Biomedical Science and Engineering, National-Yang Ming University, Taipei, Taiwan; 5 Research Institute for Microbial Disease, Osaka University, Osaka, Japan; 6 Graduate School of Life Sciences, Tohoku University, Sendai, Japan; 7 Graduate School of Frontier Bioscience, Osaka University, Osaka, Japan; 8 Center for Frontier Oral Science, Graduate School of Dentistry, Osaka University, Osaka, Japan; 9 Center of Infectious Disease and Signaling Research, National Cheng Kung University, Tainan, Taiwan; Columbia University, UNITED STATES

## Abstract

Group A *Streptococcus* (GAS) is deleterious pathogenic bacteria whose interaction with blood vessels leads to life-threatening bacteremia. Although xenophagy, a special form of autophagy, eliminates invading GAS in epithelial cells, we found that GAS could survive and multiply in endothelial cells. Endothelial cells were competent in starvation-induced autophagy, but failed to form double-membrane structures surrounding GAS, an essential step in xenophagy. This deficiency stemmed from reduced recruitment of ubiquitin and several core autophagy proteins in endothelial cells, as demonstrated by the fact that it could be rescued by exogenous coating of GAS with ubiquitin. The defect was associated with reduced NO-mediated ubiquitin signaling. Therefore, we propose that the lack of efficient clearance of GAS in endothelial cells is caused by their intrinsic inability to target GAS with ubiquitin to promote autophagosome biogenesis for xenophagy.

## Author summary

Autophagy is an intracellular bulk degradation system to survive within starved condition, which is one of the most common threats limiting organism’s expansion. By the system, cells digest their own cytoplasmic compartments that are sequestered by double membrane structure called autophagosome. It is also utilized for selective targeting of unwanted materials inside the cells including invading bacteria. This system targeting bacteria is called xenophagy, and provides “non-specialists” with innate immune system. Xenophagy is well characterized in epithelial cells since they are primary targets for invading bacteria. However, even though bacterial penetration into blood vessel could cause severe symptoms, it remains unknown weather endothelial cells retain functional xenophagy fighting against them. In this report, we showed that endothelial cells fail to suppress group A *Streptococcus* (GAS) growth due to defect in xenophagy. It is due to endothelial cell’s defect in ubiquitination of GAS, which plays a key role in target recognition during selective autophagy. Because we confirmed that endothelial cells are proficient in canonical, non-selective autophagy, our findings illustrate intrinsic defect in xenophagy as unique but general character of endothelial cells, shedding light onto cell type specificity in selective autophagy.

## Introduction

*Streptococcus pyogenes*, also known as group A *Streptococcus* (GAS), is a common human pathogen that causes a variety of illnesses, ranging from mild self-limiting infections to severe invasive diseases. Pathogenesis involves various virulence factors for adhesion, invasion, colonization, and defense against the immune system [1–4]. Although GAS is defined as an extracellular bacterium that is recognized by phagocytes through PRRs (pattern recognition receptors), triggering further immune responses [5], it can also invade eukaryotic cells, allowing the bacteria to escape from immune cell clearance and antibiotic killing [6–8]. Nonetheless, the host can eliminate internalized bacteria via xenophagy [9–11], a specialized form of the intracellular bulk degradation system known as autophagy. The xenophagy pathway is utilized not only in specialist immune cells, but also in other cell types such as epithelial cells.

Autophagy digests intracellular components to obtain minimum energy and basic building blocks to ensure cellular survival during starvation conditions. Following non-selective engulfment of cytoplasmic components by a double-membrane structure called the autophagosome, these compartments fuse with lysosomes, where the cargos are degraded by hydrolases [12,13]. Autophagy can also specifically target intracellular components including damaged organelles, protein aggregates, and invading bacteria in order to maintain cellular and systemic homeostasis. As a form of selective autophagy, xenophagy requires a mechanism for cargo recognition. In addition, xenophagy is distinguished from other forms of autophagy by its requirement for engulfment of bacteria. The GAS-containing autophagosome-like vacuoles (GcAVs), which trap invading GAS inside host cells, are often nearly 10 μm in diameter, and are thus capable of sequestering multiple bacteria; by contrast, regular autophagosomes range between 0.5 and 1.5 μm [11,14]. To build up this large structure, xenophagy requires Rab7, Rab9A, and Rab23 GTPases in addition to the core sets of autophagy genes [15,16].

The most significant difference between xenophagy and other forms of selective autophagy is that it targets intruders from outside the body. Thus, in order to understand the significance and mechanism of xenophagy, we must consider the penetration path and cell-type specificity of the invading pathogens. Most research in xenophagy has been focused on epithelial cells in the intestinal and respiratory tracts, because these cells are the primary targets of bacterial invasion into organisms. However, given that intrusion of bacteria into the cardiovascular system causes their dissemination throughout the body, leading to potentially fatal consequences, endothelial cells should also be investigated as important targets. Consistent with this idea, GAS can enter human umbilical vein endothelial cells (HUVEC) [17], and invasive GAS can survive in endothelial cells [18,19]. However, the details of bacterial fate after engulfment into endothelial cells remain poorly defined.

In this study, we found that GAS could survive and multiply in endothelial cells, whereas epithelial cells efficiently removed them via functional xenophagy. Invading GAS is decorated with the autophagosome marker LC3 in endothelial cells, but they could not be surrounded by the autophagic double-membrane structure, leading to fatal consequences. This intrinsic defect in endothelial xenophagy most likely stems from insufficient ubiquitination of invading GAS mediated by the nitric oxide (NO) pathway. Our findings regarding the cell-type specificity of xenophagy provide essential insights into the mechanisms of cellular defenses against bacterial invasion *in vivo*.

## Results

### Endothelial cells are less efficient than epithelial cells in suppressing growth of infecting GAS

**Fig 1:**
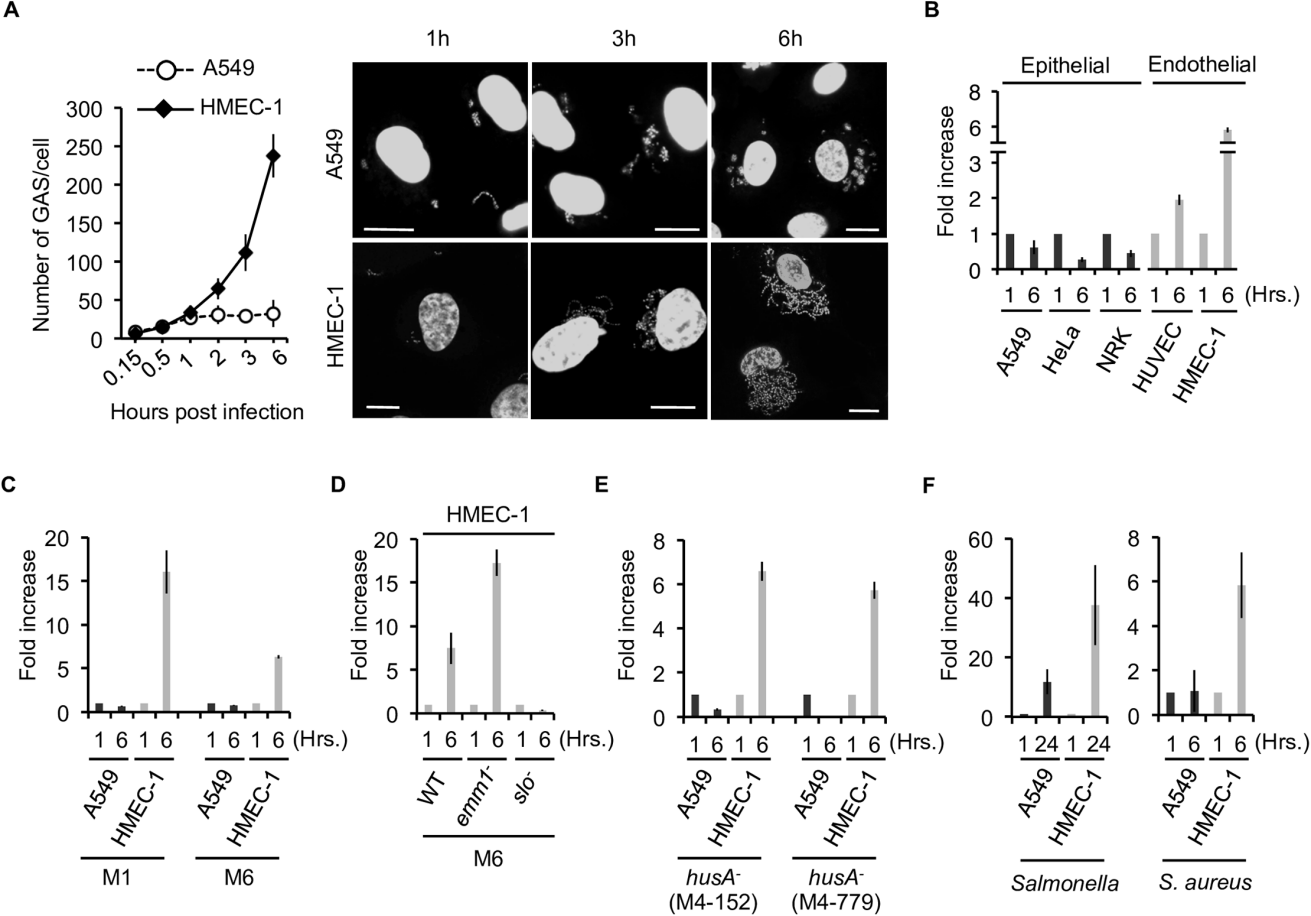
Bacteria survive and multiply in endothelial cells. **(A)** Summarized bar graph (left) showing that GAS massively proliferated in endothelial HMEC-1 cells, but not in epithelial A549 cells. Representative images (right) show DAPI staining of host nuclei and infected GAS. Scale bar, 10 μm. **(B-F)** Bacterial multiplication in the indicated cell lines at indicated time point vs. 1 h post-infection is depicted by the bar graph. GAS strain, A20 (M1 serotype) and JRS4 (M6 serotype) **(C)**, JRS4 wild type, *emm1* and *slo* mutant strains **(D),** two *hasA* mutant strains (number M4-152, M4-779) **(E)**, *Salmonella* **(F,** left**)** and *S*. *aureus* **(F,** right**)** were used for infection**.** Error bars indicate SD of three independent experiments.

To determine the fate of GAS following internalization into endothelial cells, we compared the human microvascular endothelial cell line-1 (HMEC-1) with lung epithelial A549 cells. The efficiency of GAS (NZ131, type M49) invasion was 4–5 times higher in endothelial cells (10.6±2.031 x 10^4^ cfu/ml) than epithelial cells (2.433±0.339 x10^4^ cfu/ml), so we adjusted the ratio of the MOI between endothelial and epithelial cells to 1:5 in order to monitor GAS survival following engulfment of equal numbers of GAS into both cell types. Strikingly, a time-course growth analysis of intracellular GAS revealed that GAS multiplied in HMEC-1 cells, but remained suppressed in A549 cells (Fig 1A). GAS, detected as DAPI-stained dots in the cytoplasm, increased in abundance in a time-dependent manner (Fig 1A). Majority of endothelial cells with GAS was still viable at 6 hours post infection, but eventually resulted in necrotic cell death at 24 hours (S1A–S1D Fig).

To confirm that internalized GAS is capable of replication, we recovered bacteria inside the cells and performed colony-formation assays. The results revealed an increase in the number of colonies formed by GAS recovered from endothelial cells 6 h post-infection, whereas those from epithelial cells exhibited reduced viability (Fig 1B). This expansion was not specific to HMEC-1 cells, as we observed a similar phenotype even in untransformed, primary human umbilical vein endothelial cells (HUVEC) (Fig 1B), suggesting that the insufficient GAS clearance is not the artificial event specific to immortalized cells. Furthermore, in addition to A549 cells, GAS did not grow in two other epithelial cell types (HeLa and NRK). This endothelial defect in GAS growth suppression is not specific to the M49 serotype, because HMEC1 cells, but not control A549 cells, failed to suppress expansions M1 and M6 GAS strains as well (Fig 1C).

We assumed that bacterial virulence factors might be involved in this event, and thus examined the growth of GAS harboring a mutation in *emm1* gene encoding a M protein, or *hasA* gene which is required for formation of hyaluronic acid capsule. We observed the expansion of GAS with either mutation in endothelial cells (Fig 1D and 1E), suggesting that GAS expansion in endothelial cells is not due to a lack of endothelial defense mechanism against those virulence factors. Moreover, we determined specie specificity for this defect. To this end, we infected cells with *Salmonella* and also *S*. *aureus*. Both two bacterial strains showed striking growth in endothelial cells but not in control epithelial cells, suggesting that this is not an event specific to GAS but rather a phenomenon generally observed (Fig 1F). Taken together, we concluded that endothelial cells are deficient in suppression of intracellular growth of GAS.

### Compromised autophagosome formation on GAS inside endothelial cells

**Fig 2:**
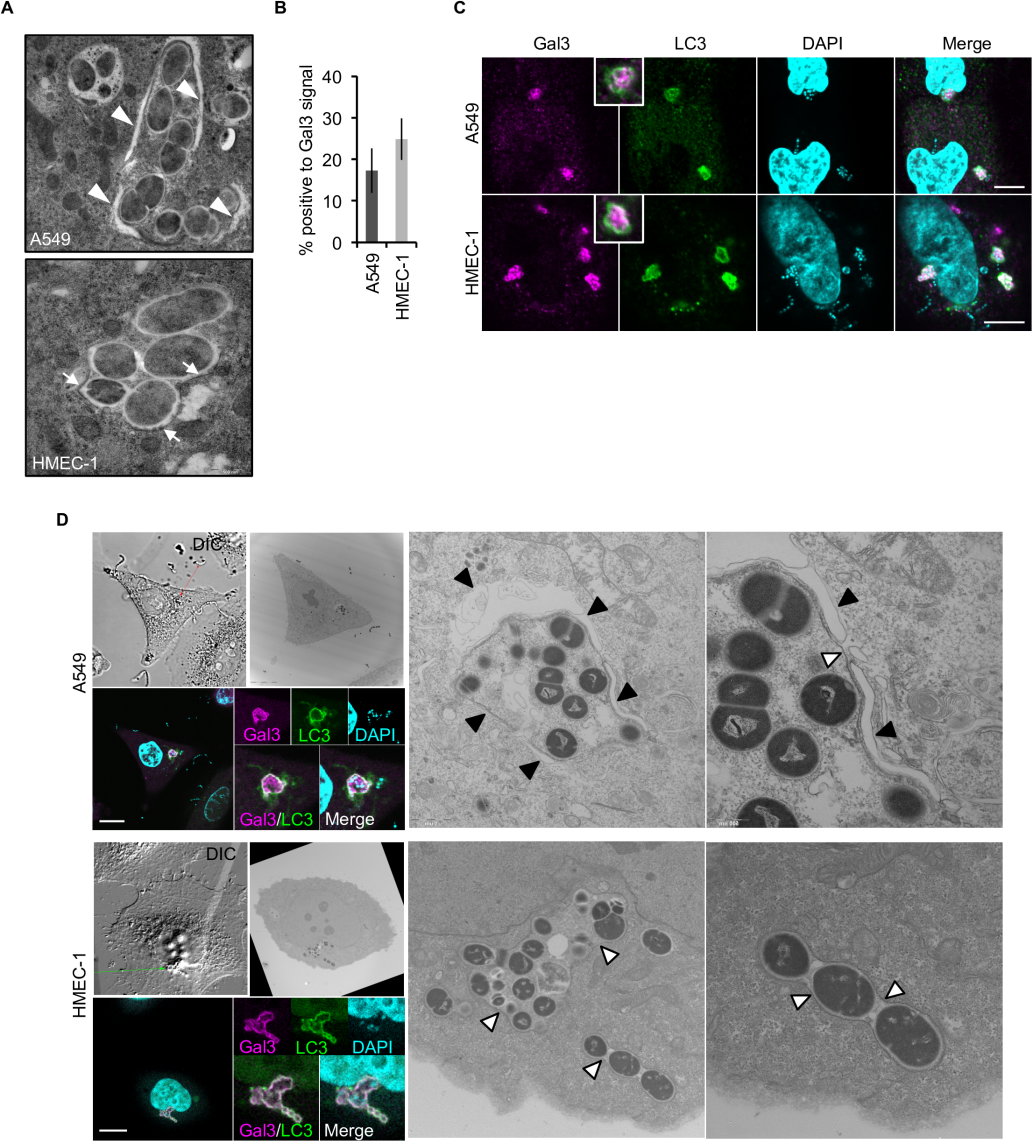
Endothelial cells are intrinsically defective in formation of double-membrane structures surrounding GAS. **(A)** Representative EM images show double-membrane structure (arrowheads) in A549 cells, but not in HMEC-1 cells. HMEC-1 cells were only positive for single membranes (arrow) at 1 h post-infection. (**B)** GAS with ruptured endosomal membranes in A549 and HMEC-1 cells were stained by anti-Gal3 antibody. Error bars indicate SD of three independent experiments. (**C)** Immunocytostaining images show LC3 signals on GAS with ruptured endosomal membranes (Gal3) at 1 h post-infection. Scale bar, 10 μm. **(D)** Representative CLEM images show LC3 (GFP) and Gal3 (Strawberry) signal on membrane structures surrounding GAS. A total of 16 (epithelial cells) and 36 (endothelial cells) GAS-containing vacuoles doubly positive for Gal3 and LC3 were observed. Black arrowheads double-membrane structure; white arrowheads, single-membrane structure. See also S2 Fig. Scale bar, 10 μm.

To obtain mechanistic insights into the failure of endothelial cells to suppress GAS growth, we studied the role of autophagy in GAS clearance in light of its significant role in GAS elimination in epithelial cells [11,15,16,20–22]. This idea is consistent with our observation that GAS with mutation in *slo* encoding Streptolysin O (SLO) did not expanded even in endothelial cells (Fig 1D), as GAS mutated in *slo* is degraded by a manner independent from autophagy [20]. To compare GAS autophagy between the two cell types, we first sought to exclude the possibility that endothelial cells have a defect in canonical autophagy induced by serum starvation. In response to starvation, endothelial cells are able to form LC3 puncta and exhibited autophagic flux at levels comparable to those in epithelial cells (S1E–S1G Fig), indicating that endothelial cells retain normal activity of canonical autophagy. Next, we focused on selective autophagy in response to GAS infection. We observed that GAS induced LC3 lipidation in a time- and infectious dose–dependent manner, as well as LC3 puncta formation in endothelial cells (S2A–S2C Fig), implying that autophagy could be induced by GAS invasion even in endothelial cells. However, using conventional EM, we rarely observed GAS surrounded by double-membrane structures in endothelial cells, whereas GAS in epithelial cells were positive for the structure (Fig 2A). To rule out the possibility that formation of the structure was simply delayed, we performed EM at later time points after infection. At these later times, we still found no isolation membrane surrounding the bacteria, and GAS continued growing in the cytoplasm of endothelial cells (S2D Fig). These results strongly suggest that endothelial cells are defective in sequestration of cytoplasmic GAS with autophagosome, and that the elevation in LC3 lipidation induced by GAS is unlikely to be related to the ability to suppress bacterial growth.

To investigate the molecular underpinnings of the less efficient xenophagy in endothelial cells, we asked whether endothelial cells were capable of detecting invading GAS. It has been suggested that invading bacteria can be recognized as invaders by the ruptured endosome surrounding them: luminal exposure of ruptured endosomes facilitates further recruitment of ubiquitin, which plays an essential role in xenophagy [23–25]. To explore this possibility, we measured GAS with Gal-3, a marker for ruptured endosome [24]. However, we observed no significant difference in the proportion of GAS with Gal-3 signals in endothelial versus epithelial cells (Fig 2B), suggesting that initial cue for xenophagy is triggered by GAS, even in endothelial cells. Moreover, we could observe overlap between the LC3 and Gal-3 signals on invading GAS, even in endothelial cells (Fig 2C). However, correlative light electron microscopy (CLEM) analysis clearly showed that those LC3 signals were not correlated with autophagosomal double membranes (Fig 2D and S3A–S3D Fig), again indicating that endothelial cells were defective in formation of autophagosomes surrounding invading GAS. Consistent with this, the number of LC3-positive GAS kept increasing over time in HMEC-1 cells, whereas their multiplication was suppressed in epithelial cells (S4A and S4B Fig), suggesting that the LC3 signals on GAS in endothelial cells did not indicate functional autophagosomes. A lack of autophagosome formation surrounding GAS is observed in primary HUVEC as well. Consistently, in HUVEC, GAS is able to be positive to both LC3 and Gal-3 signal but totally negative to autophagosomal double membrane structures (S3E Fig). Taken together, these findings indicate that endothelial cells are incompetent to suppress GAS growth due to impairment in formation of autophagosome membranes surrounding GAS with ruptured endosomes.

### Insufficient GAS elimination in endothelial cells is a consequence of less efficient ubiquitin coating

**Fig 3:**
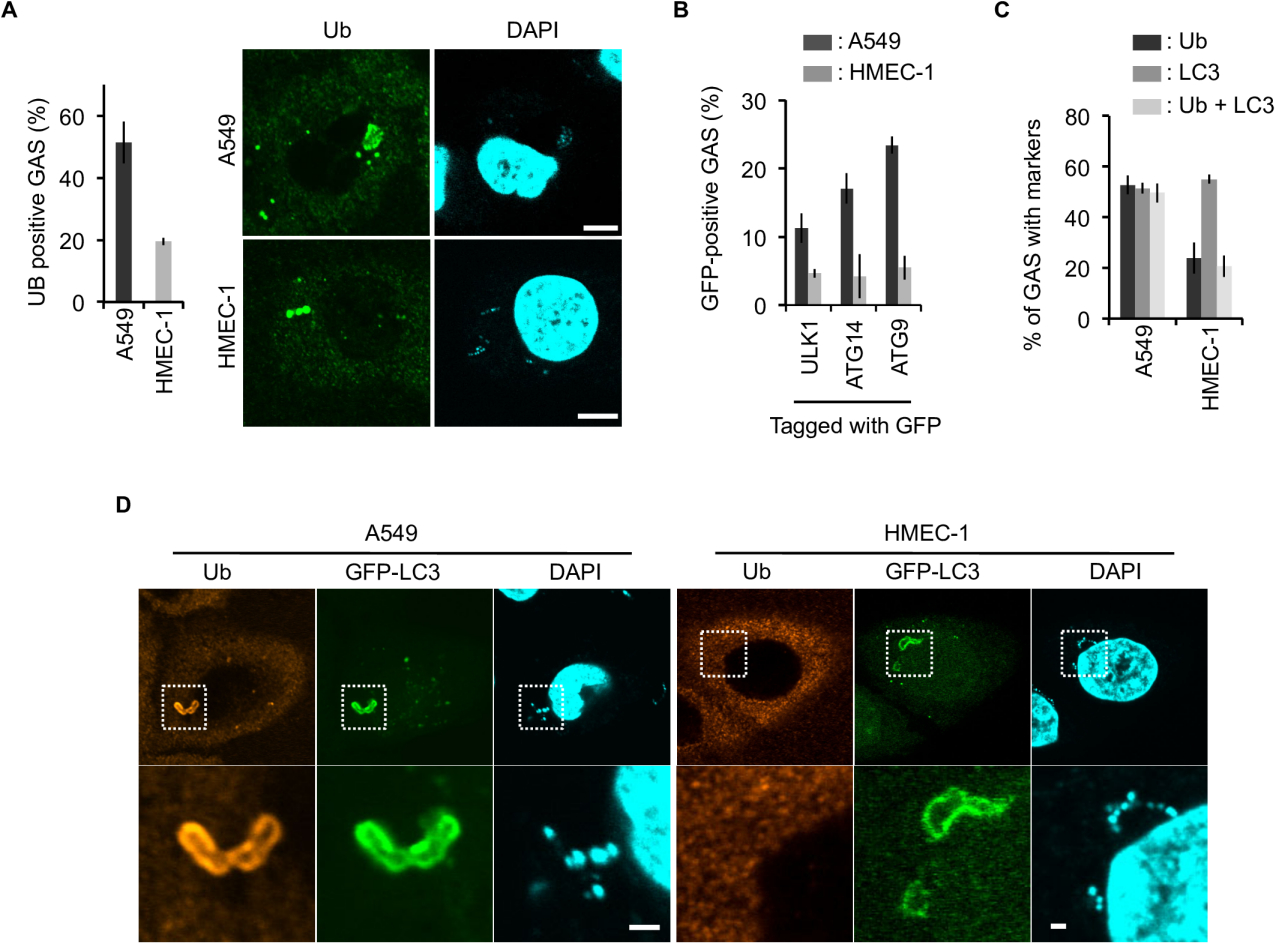
Defect in recruitment of ubiquitin- and autophagy-related proteins on GAS in endothelial cells. **(A)** Representative images of GAS (1 h post-infection) with ubiquitin signal in the indicated cell lines (right). Summarized bar graph is at left. **(B)** Percentages of GAS (1 h post-infection) with the indicated tagged GFP signals are summarized in the bar graph. **(C)** Percentage of GAS (1 h post-infection) with LC3, ubiquitin, and both signals are summarized in the bar graph. **(D)** Representative images for **(C)**. **(A, B, C)** Error bars indicate SD from three independent experiments, with > 100 cells in each sample. Scale bars, 10 μm for **A** and 1 μm for **D**.

To determine the mechanism that gives rise to defective formation of autophagosomal membrane surrounding GAS, we focused on ubiquitination, which is an essential step for initiation of selective autophagy [26–28]. Consistent with this, deregulation of ubiquitination impairs recruitments of downstream components of xenophagy [29,30]. Strikingly, we found that recruitment of ubiquitin to GAS was reduced in endothelial cells in comparison with epithelial cells (Fig 3A), correlated with defective recruitment of downstream ATG proteins. The recruitments of ULK1, ATG14, and ATG9, all involved in formation of isolation membrane and xenophagy [31,32], were significantly reduced in endothelial cells (Fig 3B and S5A Fig). Not only for GAS, we confirmed that invading *Salmonella* is targeted by ATG9 at a lower level in endothelial cells than that in epithelial cells, together indicating that endothelial cells are compromised in early step of xenophagy (S5B Fig). As expected, GAS with LC3 signals were observed regardless of ubiquitination in endothelial cells, in contrast to the case of epithelial cells, where most LC3-positive GAS were associated with ubiquitin (Fig 3C and 3D). Accordingly, we hypothesized that ineffective ubiquitination on bacteria could be the major cause of the defect in xenophagy in endothelial cells.

**Fig 4:**
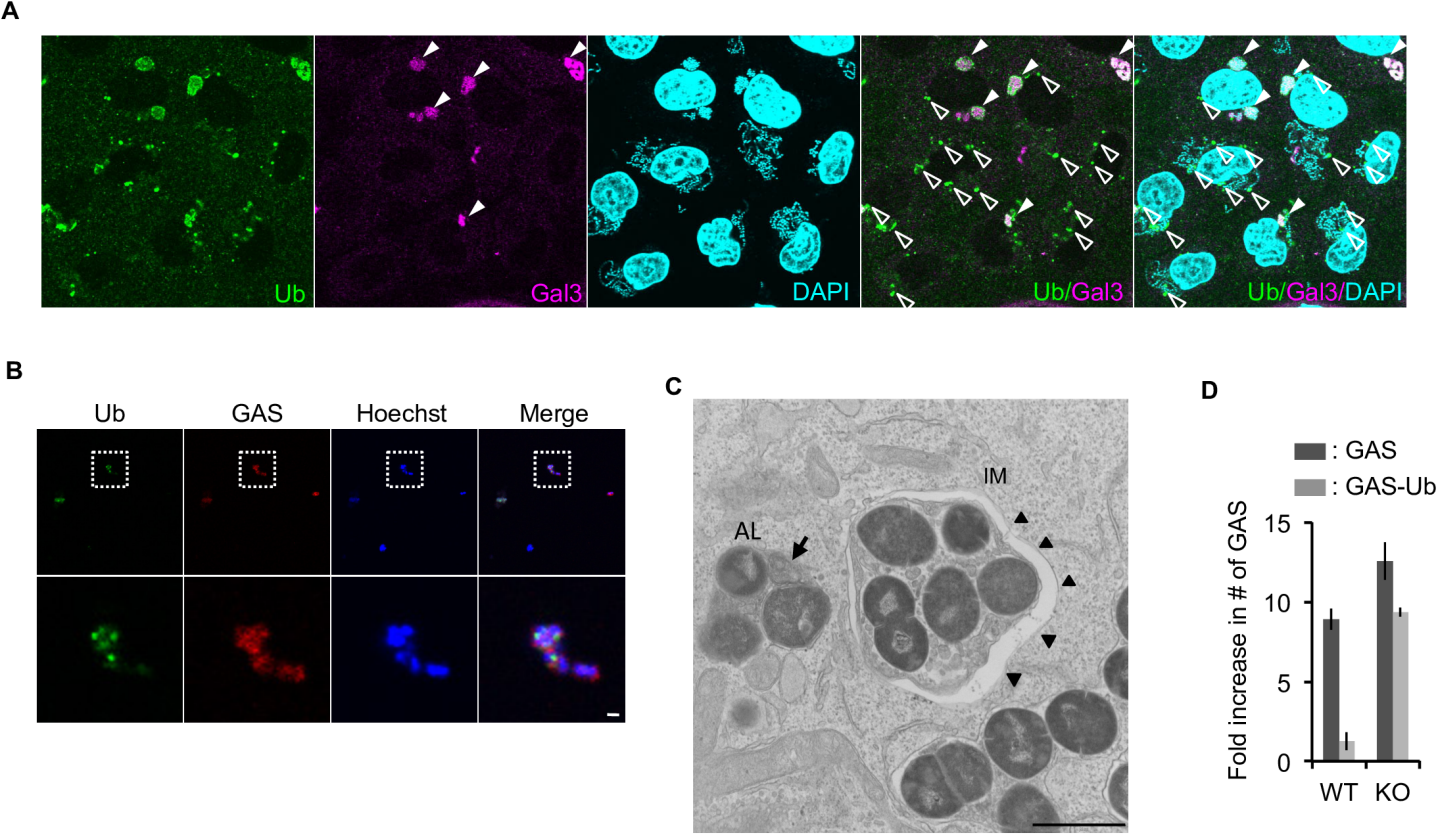
Exogenous ubiquitin coating is sufficient to eliminate GAS via xenophagy, even in endothelial cells. **(A)** *ATG*9-KO HeLa-Kyoto cells with GAS (3 h post-infection) were immunostained with anti-ubiquitin and Gal3. Filled and empty arrowheads indicate ubiquitin-positive GAS with or without Gal3 signal, respectively. **(B)** GAS harvested from host HeLa cells. Scale bars, 10 μm for **A** and 1 μm for **B**. **(C)** Representative EM images show double-membrane structure (arrowheads) in Ub-coated GAS-infected HMEC-1 cells. IM, isolation membrane; AL, autophagolysosome. Arrow indicates multiple membrane and arrowheads indicate IM. Scale bar, 1 μm. **(D)** Fold replication of GAS in wild-type or *ATG*9-KO HMEC-1 cells 6 h post-infection with naïve GAS or Ub-coated GAS. Error bars indicate SD from three independent experiments.

To directly test this hypothesis, we pre-coated GAS with ubiquitin and asked whether they would be eliminated even in endothelial cells. To generate a physiologically relevant coat of ubiquitin, we used epithelial cells as ‘craftsmen’. Notably, we used *ATG9*-knockout (KO) HeLa epithelial cells (S6A–S6C Fig) for this purpose in order to avoid degradation of the product by the functional xenophagy system in wild-type cells. Using this system, we could successfully recover GAS with a ubiquitin coat from *ATG9*-KO HeLa cells, whose integrity of ubiquitination was confirmed by immunostaining (Fig 4A and 4B). To recover intact GAS with the coat, we treated infected HeLa cells with distilled water without any detergents so that the osmotic imbalance would disrupt the plasma membrane of the host cells, concurrently enabling us to exclude the possibility that harvested GAS were surrounded by intact endosome structure that might burst under such harsh condition. When we infected endothelial cells with naïve GAS or ubiquitin-coated GAS, we found that, even in endothelial cells, GAS were engulfed by autophagosomal double membrane, and efficiently eliminated if they were coated with ubiquitin (Fig 4C and 4D). This observation strongly supports the hypothesis that the defect in xenophagy in endothelial cells results from reduced ubiquitination activity on invading GAS, rather than a deficiency in autophagosome biogenesis itself. This is consistent with our observation showing that endothelial cells retained canonical autophagic activity, comparable to the level in epithelial cells (S1A–S1C and S2A–S2C Figs). The observed clearance of ubiquitin-coated bacteria in endothelial cells requires the intact autophagic machinery, because *ATG9*-KO endothelial cells (S6D–S6F Fig) failed to suppress the replication of GAS with a ubiquitin coat (Fig 4D). In addition, under naïve GAS infection, bacterial growth was slightly exacerbated in *ATG9*-KO endothelial cells in comparison with wild-type cells (Fig 4D and S6F Fig), suggesting that autophagic machinery does indeed help endothelial cells to defend against bacterial infection, even though the resultant suppression of GAS growth is far from sufficient.

### Defective response of nitrated nucleotide–mediated ubiquitination on GAS in endothelial cells

To further explore these findings, we sought to determine whether the defect in ubiquitination was functionally associated with the nitric oxide (NO)-mediated signaling cascade. Recent work showed that this pathway facilitates ubiquitination and selective clearance of intracellular GAS in macrophages [33]. GAS invasion induces NO synthase (NOS)-mediated NO production, resulting in generation of 8-nitroguanosine 3′,5′-cyclic monophosphate (8-nitro-cGMP, an endogenous derivative of cGMP). It is followed by S-guanylation on cysteine residues of targets at the bacterial surface. S-guanylation promotes ubiquitination of the bacteria (summarized in S7A Fig). In this cascade, L-NMMA, a NOS inhibitor, or NaHS, an 8-nitro-cGMP eliminator, can inhibit ubiquitination and degradation of bacteria [33].

**Fig 5:**
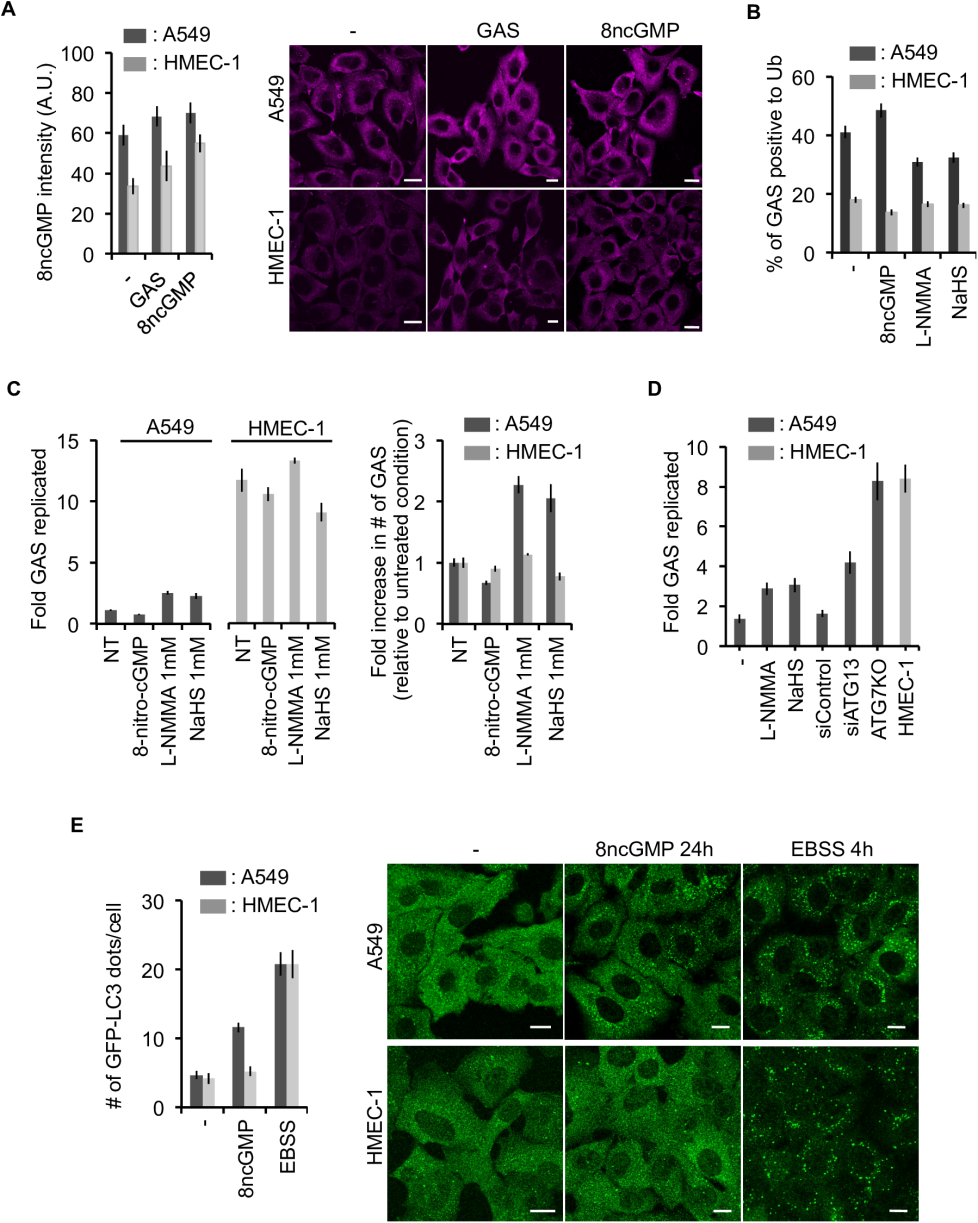
Deficiency of 8-nitro-cGMP–mediated autophagy of GAS in endothelial cells. **(A)** Summarized bar graph (left) and representative immunostaining images (right) of endogenous levels of 8-nitro-cGMP induced by GAS infection. Cells treated with 8-nitro-cGMP (100 μM, 24 h) were used as positive controls. Scale bar, 10 μm. **(B)** Frequency of GAS positive for ubiquitin was measured in the presence of inhibitors (NaSH, 1mM or L-NMMA, 1 mM) or an activator (8-nitro-cGMP). Bar graph of a parallel colony formation experiment is shown on **(C)**. Left panel shows fold replication of GAS relative to initial number of infecting GAS, and right panel shows numbers of GAS relative to each non-treatment experiment. **(D)** Fold replication of GAS with the inhibitor, siRNA for ATG13, or knockout of ATG7 was examined by colony formation assay. **(E)** Formation of LC3 puncta induced by 8-nitro-cGMP. EBSS (4 h) was used as a positive control. Scale bar, 10 μm. Error bars indicate SD from three independent experiments.

To determine whether the NOS-ubiquitin pathway is intact in endothelial cells, we measured endogenous 8-nitro-cGMP levels in these cells, and found that they had an intrinsically lower level of 8-nitro-cGMP than epithelial cells (Fig 5A). Additionally, GAS infection–induced 8-nitro-cGMP failed to compensate for this defect, whereas exogenous introduction of 8-nitro-cGMP increased its level inside the cell, even in endothelial cells. This observation indicates that endothelial cells are intrinsically deficient in both production and induction of 8-nitro-cGMP. This conclusion was further confirmed by treating cells with two inhibitors, L-NMMA and NaHS. These experiments showed that the poor ubiquitination of GAS in endothelial cells was not altered by the inhibitors, whereas ubiquitination in epithelial cells was significantly reduced (Fig 5B). Notably, this is not merely due to low levels of 8-nitro-cGMP in endothelial cells, because introduction of exogenous 8-nitro-cGMP failed to restore GAS ubiquitination in these cells, implying that, in addition to an intrinsically low level of 8-nitro-cGMP, endothelial cells lack a mechanism to facilitate ubiquitination of GAS mediated by this compound (Fig 5B). Consistent with this, endothelial cells did not exhibit any detectable increase in GAS growth upon treatment with L-NMMA or NaHS (Fig 5C and 5D, S7B–S7D Fig), whereas GAS replication was elevated in epithelial cells treated with those inhibitors or harboring silencing/knockout of autophagy genes, supporting the idea that endothelial cells are deficient in bacterial clearance via NOS–ubiquitin pathway.

Taken together, our data suggests that deficiency in the NOS–ubiquitin pathway in endothelial cells is at least partially responsible for their diminished ability to defend themselves against GAS. Additionally, our results also imply that endothelial cells not only fail to form GcAVs, but also cannot induce formation of regular autophagosomes in response to 8-nitro-cGMP. Formation of LC3 puncta formation could be induced by 8-nitro-cGMP in epithelial cells, but not in endothelial cells (Fig 5E), indicating that the lack of the NOS-dependent induction of autophagy in endothelial cells is linked to a wide range of physiological outcomes related to autophagy.

## Discussion

In this report, we showed that xenophagy is not equally efficient among different cell types, raising the question of why endothelial cells do not retain this vital ability to defend themselves against bacteria. We found that GAS efficiently replicates in endothelial cells at a rate comparable to that of *in vitro* GAS growth [34,35], indicating that the bacteria are essentially growing freely. We speculate that this situation evolved because robust activation of the NO pathway in endothelial cells could cause a lethal side effect on cardiovascular systems that would outweigh the beneficial effects of activating xenophagy. Essentially, constitutive releases of NO from endothelial cells via endothelial NOS (eNOS) serves a protective role in cardiovascular homeostasis by relaxing blood vessel pressure [36]. However, excess induction of NO, which activates the 8-nitro-cGMP pathway and xenophagy, could deregulate blood vessel pressure. In fact, robust NO induction by bacterial infection or LPS causes hypotension and sepsis, a phenomenon mediated by inducible NOS (iNOS) [37]. We found that endothelial cells have low levels of 8-nitro-cGMP and do not efficiently ubiquitinate invading GAS in response to 8-nitro-cGMP. This is consistent with the fact that NO production in endothelial cells is directly connected to that of NO in vascular muscle [36], creating a situation more sensitive than that in epithelium. Therefore, we speculate that, given the need for tight regulation of this pathway, endothelial cells had to abandon NO-mediated induction of xenophagy.

We found that exogenous coating of GAS with ubiquitin was sufficient to induce their clearance, even in endothelial cells, in a manner dependent on the autophagic machinery. This observation is in line with the recently established consensus that ubiquitination plays an essential role in cargo recognition in selective autophagy. Thus, endothelial cells lack a key ability to target invading bacteria. What, then, is the underlying mechanism explaining the failure of endothelial cells to perform this function? We suspect that this deficiency is not merely due to their poor NO-pathway response. We showed that in epithelial cells, NO pathway inhibitors only partially increased bacterial growth, to levels lower than those observed in *ATG7*-KO cells (Fig 5D). This was not simply because of an insufficient suppression of the NO-mediated pathway, as much higher doses of inhibitors did not further increase GAS growth (S7E Fig), It suggests that inhibition of epithelial NO-mediated ubiquitination pathway only partially recapitulates the xenophagy defect in endothelial cells, which is supposed to be caused by insufficiency in multiple systems. We assume that it is mediated by ubiquitin targeting system involving specific E3 ligase(s) [38–40]. Identification of these pathways in endothelial cells is a high priority because it may facilitate development of a novel approach to fighting GAS, in particular to alleviate their expansion in cardiovascular system and prevent life-threatening bacteremia, which is often exacerbated by antibiotic resistance or delay in a treatment.

In this study, we found that invading GAS is often positive for the autophagosome marker LC3. However, we found that these LC3 signals are not related to functional autophagy. Considering the poor ability of endothelial cells to eliminate GAS, this previously unknown autophagy-independent coating of GAS with LC3 may play only a minor role in innate immunity, whereas *ATG9* knockout results in slightly exacerbated GAS growth, even in endothelial cells. Because LC3 lipidation can be induced by GAS infection in endothelial cells (S2A–S2C Fig), LC3 molecules on GAS are likely to be in the lipidated form. We speculate that the autophagic machinery facilitates LC3 lipidation for this event trying to limit their growth by autophagy-independent mechanism although it remains unclear how lipidated LC3 is recruited to invading GAS. It could be related to another autophagy-independent role for autophagy-related genes, as represented by LC3-associated phagocytosis (LAP), although it is less effective than LAP.

In summary, we showed that endothelial cells are not capable of carrying out xenophagy due to an intrinsic defect in ubiquitin-targeting system. Because ubiquitination is a universal key event in selective autophagy, further characterization of the mechanism may provide perspective and valuable insights into host defenses, as well as alleviate a wide range of diseases suppressed by selective autophagy.

## Materials and methods

### Cell culture

Human microvascular endothelial cell line-1 (HMEC-1) [41] (obtained from the Centers for Disease Control and Prevention, USA) was cultured in endothelial cell growth medium M200 (Cascade Biologics) supplemented with 10% fetal bovine serum (FBS), 1 μg/ml hydrocortisone, 10 ng/ml epidermal growth factor, 3 ng/ml basic fibroblast growth factor, and 10 μg/ml heparin. Human umbilical vein endothelial cells (HUVEC, gift from Dr. H. Y. Lie) were maintained in M200 medium containing 10% FBS as HMEC-1 cells. A549 (laboratory stock), HeLa (laboratory stock), and NRK (normal rat kidney epithelial, laboratory stock) cells were maintained in Dulbecco’s modified Eagle’s medium (DMEM) supplemented with 10% FBS. Cells were cultured at 37°C in 5% CO_2_.

### Bacterial culture

*Streptococcus pyogenes* strain NZ131 (type M49) is a gift from Dr. D. R. Martin (New Zealand Communicable Disease Center, Porirua). A20 (type M1), JRS4 (type M6) wild type, *emm1* mutant, *slo* mutant, *hasA* mutant GAS strains and standard strain of *Staphylococcus aureus* (ATCC 25923) are provide from Dr. J. J. Wu. *Salmonella enterica* serovar Typhimurium SR-11 x3181 was used for infection. Bacteria were grown overnight at 37°C in tryptic soy broth with 0.5% yeast extract (TSBY) for GAS and *S*. *aureus*, and LB broth for *Salmonella*, and then transferred to fresh broth for 3 h. The culture was centrifuged and suspended in phosphate-buffered saline (PBS), followed by measurement of cell concentration as (0.2 OD_600_ = 1 × 10^8^ cfu/ml, confirmed by plating). This procedure for bacterial preparations was used for all infectious experiments in this study. For heat inactivation, the suspended bacteria were treated at 70°C for 30 min.

### Bacterial infection

A monolayer of cells was plated in 24-well or 6-well plates and incubated overnight. The prepared bacteria were directly added to the wells at a multiplicity of infection (MOI) of 1, 5, 10, or 25, and then centrifuged at 500 *g* for 5 min to ensure simultaneous infection of cells. In one set of experiments, the ratio of GAS and *S*. *aureus* infectious MOI between epithelial cells and endothelial cells was maintained at 5:1, due to the 5-fold higher internalization efficiency in endothelial cells relative to epithelial cells. *Salmonella* was used at MOI of 100 for both cell type infections. The cell and bacteria mixture was incubated at 15 min for *Salmonella* and 30 min for GAS and *S*. *aureus*. After incubation, the cell culture was washed twice with PBS to remove unattached bacteria, and then fresh medium containing 100 μg/ml gentamicin was added to kill the remained extracellular bacteria. After various time periods, cells were collected for individual experiments.

### Immunofluorescence staining

Cells seeded at 6 × 10^4^/well in 24-well plates with cover glasses were cultured overnight and infected with GAS according to the infection protocol. Cover glasses were coated with cellular matrix (Cellmatrix type I-C, 100 μg/ml, 37°C, 30 min) in advance. At various time points post-infection, the cells were fixed with 4% paraformaldehyde (PFA), permeabilized with 50 μg/ml digitonin, and stained with anti-GAS (gift from Dr. J. J. Wu), anti-galectin-3 (M3/38, Santa Cruz Biotechnology), anti-LC3 (PM036, MBL), anti-FK2 (BML-PW8810, ENZO), anti-LAMP-1 (H4A3, Santa Cruz Biotechnology), or anti-8-nitro-cGMP (1G6) antibodies (gift from Dr. T. Akaike), followed by staining with secondary antibodies conjugated with Alexa Fluor 488 or 568 and imaging on a confocal microscope (FV1000; Olympus).

### Transfection

Cells at 5 × 10^4^/well in 24-well plates were incubated overnight and transfected for 4 h with 1 μg of pEGFP-LC3 or GFP-ATGs (pEGFP-ULK-1, pEGFP-ATG14, pEGFP-ATG9) plasmid DNA using Lipofectamine 2000 reagent (Invitrogen, Carlsbad, CA, USA) in OPTI-MEM medium plus complete culture medium. After incubation at 37°C for 20 h, cells were prepared for infection.

### siRNA knockdown

Control and ATG13 siRNA (sense 5'- GAGUUUGGAUAUACCCUUUdTdT -3' and anti-sense 5'- AAAGGGUAUAUCCAAACUCdGdT -3') were purchased from Sigma-Aldrich. Cells for siRNA transfection were prepared at 1 × 10^4^/well in 24-well plates and incubated overnight. The siRNAs (20 nM) were mixed with Lipofectamine RNAiMAX reagent (Invitrogen) in OPTI-MEM medium and added to the cell culture medium. After 4 h incubation, the medium was replaced with fresh medium. After 20 h culture, a second transfection was performed using the same protocol. Forty-eight hours after the second transfection, the cells were infected with GAS or collected for western blot assays to confirm efficient knockdown of ATG13 and measure autophagic flux.

### Generation of CRISPR-Cas9 ATG7 and ATG9 knockout cell lines

To generate knock out cell lines of autophagy-related genes, we utilized the clustered regularly interspaced short palindromic repeats (CRISPR)-Cas9 system [42]. We designed RNA-guided Cas9 targeting the first exons of the human *ATG7* and *ATG9* genes. The specific recognition sequences of the 20 bp before the protospacer adjacent motif (PAM) in each construct were as follows: ATG7, 5’- CACCGAAATAATGGCGGCAGCTACG-3’; ATG9, 5’-CACCCCCTGGGGGTGAATCAC TAT-3’. These guide oligos, with *Bbs*I restriction sites at both ends, were annealed with their anti-sense oligos and inserted downstream of U6 promoter in vector pSpCas9(BB)-2A-GFP (pX458) [43] (purchased from Addgene); the resultant plasmids were used for transfection. Single-cell sorting was performed after a 48-h transfection. After 1-week culture of single cells with antibiotics, fresh medium was added to support growth for 1 more week to allow colony formation. Genomic DNA of each clone was extracted, and the target gene was confirmed by sequencing using the following primers; ATG7, Fw 5’-GTCGACGTTCTGGAGATCTGTTTCACAACG-3’ and Re 5’-GAATTCTGGGATCAAAAAGTCAGGAAG-3’; ATG9, Fw 5’-ATATGTCGACCAGGATGAGCTCCATTCCCGT-3’ and Re 5’-ATATGAATTCCAGCCCCCAACAAAGGGACAG-3’.

### Western blot

Cells were seeded at 8 × 10^4^ cells/well in 24-well plates, incubated, overnight, and then infected with GAS. At various times post-infection, cells were lysed with lysis buffer containing protease inhibitor mixture, followed by denaturation at 95°C in sample buffer, SDS-PAGE, and immunoblotting using rabbit anti-LC3 (PM036, MBL), anti-p62 (PM045, MBL), anti-ATG13 (SAB4200, Sigma-Aldrich), anti-ATG7 (013–22831, Wako), and mouse anti-GAPDH antibodies (Millipore).

### Conventional electron microscopy

Cells were infected with GAS at MOI of 25 and 5 for 1 h and Ub-coated GAS for 2 h. Cell pellets were gently collected by trypsinization and resuspended in phenol red–free medium containing 5% FBS and 40% Dextran T2000. Samples were kept on ice prior to high-pressure fixation using a High Pressure Freezer (Leica EM HPM100). After fixation, the specimens underwent freeze-substitution under low temperature and embedding in plastic (Epon812, TAAB Laboratories Equipment, Aldermaston, UK). Ultrathin sections (70 nm thick) were stained with saturated uranyl acetate and Reynolds lead citrate solution. Micrographs were acquired on a JEOL JEM-1011 transmission electron microscope (JEM-1011, JEOL, Tokyo, Japan).

### Correlative light microscopy–electron microscopy (CLEM)

Cells stably expressing GFP-LC3 and mStrawberry-galectin3 were cultured on glass-bottom dishes with a grid pattern (P35G-2-14-C-GRID; MatTek, Ashland, MA, USA) and infected for 1 h with GAS at an MOI of 25 for A549, and 5 for HMEC-1 and HUVEC cells. The cells were fixed with 4% formaldehyde in HEPES buffer (30 mM HEPES, 100 mM NaCl, 2 mM CaCl_2_, pH 7.4), and 1 μg/ml DAPI for 30 min at room temperature, washed in HEPES buffer, and observed using a confocal microscopy (FV1000; Olympus). After marking the locations of the target cells, the same specimens were further incubated with 2% formaldehyde and 2.5% glutaraldehyde in HEPES buffer at 4°C overnight. After three washes, the samples were post-fixed with 1% osmium tetroxide and 0.5% potassium ferrocyanide in HEPES buffer for 1 h, washed three times in distilled water, dehydrated in ethanol, and embedded in Epon812 (TAAB Laboratories Equipment, Aldermaston, UK). Ultrathin sections (70 nm thick) were stained with saturated uranyl acetate and Reynolds lead citrate solution. Micrographs were acquired on a JEOL JEM-1011 transmission electron microscope.

### Preparation of ubiquitin-coated GAS

After overnight culture, *ATG9*-KO HeLa epithelial cells (4 × 10^5^ cells/well in 6-well plates) were infected with GAS for 30 min at an MOI of 25, as described in the infection protocol. Gentamicin was added to the culture medium to kill extracellular bacteria. At 3 h post-infection, GAS replicated in the cytoplasm of *ATG9*-KO HeLa epithelial cells. To obtain intracellular GAS from infected host cells, the cells were treated with distilled water, causing an osmotic imbalance that disrupted the plasma membranes of host cells and the endosomal membranes surrounding the GAS. Bacterial number was determined before next infection (average ~10^7^ cfu/ml). No centrifugation was performed in order to avoid aggregation between cell debris and Ub-coated GAS. For ubiquitin staining of GAS, the bacteria were attached to poly-L-Lysine–coated cover glasses and fixed with 4% PFA, and then subjected to immunofluorescence staining with anti-FK2 and anti-GAS antibodies.

For conventional EM sample, HMEC-1 cells were infected with Ub-coated GAS at an MOI of 5 (25 μl of the 1 ml lysate: expected bacterial number, 2.5x10^5^ cfu) for 2 h. Samples were fixed and prepared for convention EM observation.

For the xenophagy rescue experiment, wild-type and *ATG9*-KO endothelial cells (1 × 10^5^ cells/well, in 24-well dish) were infected with Ub-coated GAS at an MOI of 1 (10 μl of the 1 ml lysate: expected bacterial number, 10^5^ cfu). GAS prepared in regular culture were used as controls. Colony formation assays were performed at 1 and 6 h post-infection. The fold increase in GAS number, reflective of replication, was calculated as the bacterial number at 6 h normalized against the number at 1 h.

### Colony formation assay

Cells were infected with bacteria as described in the infection protocol. At the indicated time point post-infection, bacteria-infected cells were washed twice with PBS and lysed in sterile H_2_O, 1 ml/well (24-well plates). After serial dilution with PBS, the bacteria-containing PBS was plated on TSBY or LB agar plates. Colonies were counted after 24 or 48 h incubation at 37°C. Bacterial number determined 1 h post-infection was interpreted as the number of internalized GAS. The fold increase in bacterial number was calculated as the number at later time point normalized against the number of internalized bacteria. Each colony-forming assay was performed at least three times.

## Supporting information

S1 Fig GAS-infected endothelial cells undergo necrotic cell death at late time post infection, even they retain normal activity of canonical autophagy, comparable to that of epithelial cells. HMEC-1 cells were infected with GAS for 1 h and then treated with gentamicin to kill extracellular bacteria. At 6, 12 and 24 h post-infection, the cell cytotoxicity was determined using trypan blue staining **(A)** and flow cytometry in which non-fixed cells were stained with propidium iodide (PI) **(B and D)**. Positive control was collected from fixed and non-infected cells. **(C)** At various time points post-infection, GAS-infected cells were prepared for caspase-3 protein detection by western blot analysis. **(D)** GAS-infected cells were collected at 24 h post-infection. Z-VAD (25 and 50 μM) was added to the infected cells after infection for 1 h. **(E and F)** A549 and HMEC-1 cells were starved in EBSS medium, and collected samples were stained with anti-LC3 antibody at the indicated time points. Images were acquired by confocal microscopy. Scale bar, 10 μm. Formation of LC3 puncta is depicted by the bar graph. Data represent the means ± SD from three independent experiments. **(G)** Cells were treated with 10% FBS complete medium or EBSS medium with or without bafilomycin A1 (100 nM) for 2 h, and then subjected to detect protein levels of LC3 and GAPDH by western blot analysis. The data show that there was no difference in autophagic flux between two cell types.
(TIF)

S2 Fig GAS infection induces LC3 puncta formation and lipidation, but not formation of double-membrane structure surrounding GAS in endothelial cells. **(A)** HMEC-1 cells were infected with GAS at MOI = 1, 5, 10, and 25, or heat-killed GAS at MOI = 25, for 2 h. **(B)** Cells were infected with GAS at MOI = 25 and collected at the indicated time points post-infection. Gentamicin was added to kill extracellular bacteria 30 min after infection. Samples were collected for western blot analysis to detect LC3-I/II conversion. **(C)** GFP-LC3–expressing HMEC-1 cells were infected with GAS at MOI = 5 for various times and then observed by fluorescence microscopy. The proportion of cells with GFP-LC3 puncta is shown as a percentage of total GFP-expressing and GAS-infected HMEC-1 cells. Scale bar, 10 μm. **(D)** HMEC-1 cells were infected with GAS for 1 h, and then treated with gentamicin to kill extracellular bacteria. Cells were collected at the indicated time points post-infection and fixed for electron microscopy. White arrowheads indicate GAS within vesicles at early stages, and black arrows indicate GAS in the cytoplasm in late stage. No isolation membrane was detected at any time point post-infection. GAS division occurs at all stages post-infection. Scale bar, 5 μm for upper and 1 μm for below.
(TIF)

S3 Fig LC3 and Gal3-positive GAS is not surrounded by double membrane structure in endothelial cells. **(A-D)** Representative images of correlative light electron microscopy of GAS-infected cells. GFP-LC3 and Strawberry-Gal3 stably expressing A549 cells (**A** and **B**), HMEC-1 cells (**C** and **D**) and HUVEC cells (**E**) were cultured on gridded-glass bottom dishes, and then infected with GAS for 1 h. Cells were fixed and stained with DAPI for confocal microscopy. GFP-LC3 and Strawberry-Gal3 double-positive GAS were selected as targets for transmission electron microscopy. Black arrowheads indicate isolation membrane (double membrane structure), black arrows indicate multiple membrane structures inside the LC3/Gal3-decorated single membrane indicated by white arrowheads.
(TIF)

S4 Fig LC3 and/or LAMP1-positive GAS multiplies more in endothelial cells than endothelial cells. **(A)** The defect in GAS clearance in endothelial cells is correlated with accumulation of LC3- and LAMP1-positive GAS. Both A549 and HMEC-1 cells were positive for LC3 and LAMP1. At 1 h post-infection with GAS, cells were fixed and immunostained with anti-LC3 and anti-LAMP1 antibodies. Scale bar, 10 μm. **(B)** Intracellular GAS with LC3 (Top) or LAMP1 (bottom) were counted at the indicated time points post-infection. All quantitative data represent means ± SD from three independent experiments; more than 100 cells were counted in each sample.
(TIF)

S5 Fig Recruitment of autophagy-related proteins to bacteria. Cells with ectopic expression of indicated GFP-tagged proteins were infected with GAS (**A**) or *Salmonella* (**B**) for 1 h, and then examined for GFP signal on GAS within their cytoplasm. Images were acquired by confocal microscopy. Scale bars, 10 μm. Percentages of ATG9-GFP positive *Salmonella* were shown in (**B**). All quantitative data represent means ± SD from three independent experiments.
(TIF)

S6 Fig Generation of *ATG9* knockout cell line using the CRISPR-Cas9 system. **(A)** Isolated HeLa-Kyoto cells harbor an insertion at the indicated locus in the first exon of *ATG9*. PAM sequence and recognition sequence are labeled in blue and green, respectively. **(B)** Autophagic flux was measured by p62 and LC3 degradation under nutrient-replete or starvation conditions. We observed no induction of LC3 II formation, and accumulation of p62, under starved conditions in *ATG9*-KO cells. This phenotype was rescued by ectopic expression of full-length ATG9. **(C)** HeLa-Kyoto *ATG9*-KO cells exhibited an increase GAS growth. Colony-forming assay (CFA) was performed at 1 and 6 h post-infection. Fold replication of GAS was calculated by comparison of GAS number at 6 h vs. 1 h. Error bars indicate SD from three independent experiments. **(D)** Isolated HMEC-1 cells contain one-nucleotide insertion at the indicated locus on the first exon of *ATG9* gene. The PAM and recognition sequence are labeled in blue and green, respectively. **(E)** The *ATG9*-KO cell line exhibited no autophagic flux of p62 and a lack of LC3 lipidation. This phenotype was rescued by ectopic expression of full-length ATG9. **(F)** *ATG9*-KO HMEC-1 cells exhibited only a subtle increase in GAS growth relative to that in control wild-type cells. CFA was performed at 1 and 6 h post-infection. Fold replication of GAS was calculated by comparison of GAS number at 6 h vs. 1 h. Error bars indicate SD from three independent experiments.
(TIF)

S7 Fig Pathway for 8-nitro-cGMP mediated autophagy of GAS. **(A)** GAS infection induces intracellular nitric oxide (NO), which is a short-lived reactive molecule that can readily be combined with cGMP to form 8-nitroguanosine 3′,5′-cyclic monophosphate (8-nitro-cGMP). Elevated levels of endogenous 8-nitro-cGMP can increase formation of LC3 puncta and autophagy under unstressed conditions. Furthermore, this endogenous nitrated nucleotide can also modify Cys residues on GAS surface molecules by S-guanylation, which promotes ubiquitination and contributes to bacterial clearance by xenophagy. L-NMMA inhibits suppression of nitric oxide synthase (NOS) activity. NaHS provides sulfhydryl anion HS^-^ to degrade 8-nitro-cGMP into 8-SH-cGMP. **(B)** A549 cells were transfected with negative control siRNA or siRNA against Atg13 for 4 h, and then the medium was replaced prior to overnight culture. A secondary transfection was performed using the same protocol. Forty-eight hours after secondary transfection, cells were treated with complete DMEM medium or EBSS medium, with or without BafA1, for 2 h. Cell pellets were collected for determination of ATG13 (anti-ATG13 antibody, SAB4200, Sigma-Aldrich), p62, and LC3 protein levels by western blot assay. **(C)** When the CRISPR-Cas9 system was used to edit the first exon of the *ATG7* gene, there was only one thymine insertion at nucleotide position 282 (red). PAM sequence and recognition sequence are labeled in blue and green, respectively. **(D)** Autophagic flux was detected by western blotting for p62 and LC3 II form under nutrient-replete or starvation conditions in cells treated or not treated with BafA1. The protein level of ATG7 was also confirmed by western blotting. No formation of LC3 II or change in p62 levels was observed in BafA1-treated *ATG7*-KO cells. **(E)** A high dose of L-NMMA is not necessary for inhibition of NOS in GAS-infected A549 cells. CFA was performed with or without drug treatments (8-nicro-cGMP, 100 μM; L-NMMA, 1 or 10 mM). Data represent means ± SD from three independent experiments.
(TIF)
