# Supplementary figures and images for: Endothelial cells are intrinsically defective in xenophagy of Streptococcus pyogenes
Source: PLoS Pathog. 2017 Jul 6;13(7):e1006444. doi: 10.1371/journal.ppat.1006444 (PMC5500369; doi:10.1371/journal.ppat.1006444)

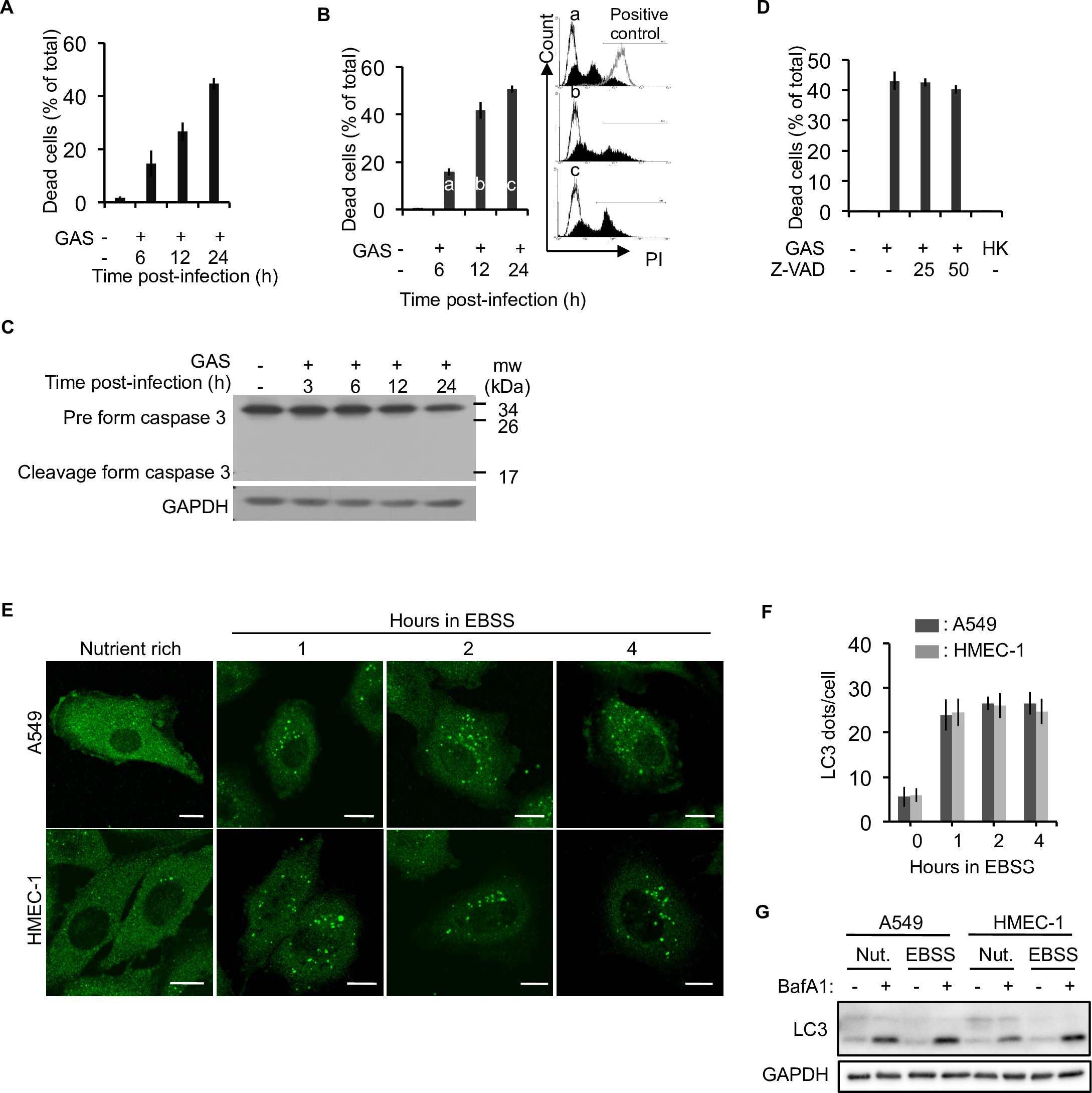

Supplement: S1 Fig — HMEC-1 cells were infected with GAS for 1 h and then treated with gentamicin to kill extracellular bacteria. At 6, 12 and 24 h post-infection, the cell cytotoxicity was determined using trypan blue staining (A) and flow cytometry in which non-fixed cells were stained with propidium iodide (PI) (B and D). Positive control was collected from fixed and non-infected cells. (C) At various time points post-infection, GAS-infected cells were prepared for caspase-3 protein detection by western blot analysis. (D) GAS-infected cells were collected at 24 h post-infection. Z-VAD (25 and 50 μM) was added to the infected cells after infection for 1 h. (E and F) A549 and HMEC-1 cells were starved in EBSS medium, and collected samples were stained with anti-LC3 antibody at the indicated time points. Images were acquired by confocal microscopy. Scale bar, 10 μm. Formation of LC3 puncta is depicted by the bar graph. Data represent the means ± SD from three independent experiments. (G) Cells were treated with 10% FBS complete medium or EBSS medium with or without bafilomycin A1 (100 nM) for 2 h, and then subjected to detect protein levels of LC3 and GAPDH by western blot analysis. The data show that there was no difference in autophagic flux between two cell types. (TIF) [file ppat.1006444.s001.tif]

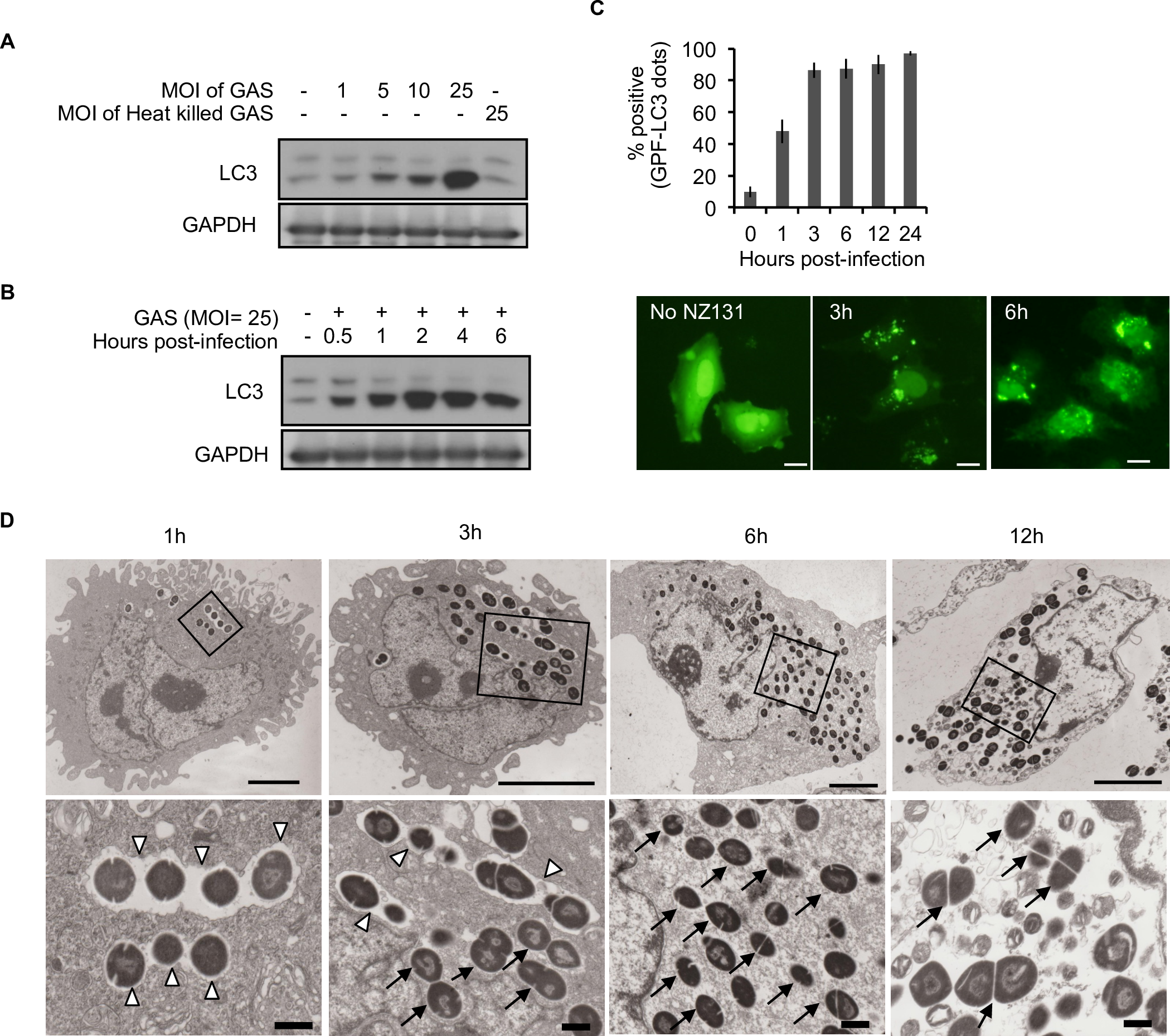

Supplement: S2 Fig — (A) HMEC-1 cells were infected with GAS at MOI = 1, 5, 10, and 25, or heat-killed GAS at MOI = 25, for 2 h. (B) Cells were infected with GAS at MOI = 25 and collected at the indicated time points post-infection. Gentamicin was added to kill extracellular bacteria 30 min after infection. Samples were collected for western blot analysis to detect LC3-I/II conversion. (C) GFP-LC3–expressing HMEC-1 cells were infected with GAS at MOI = 5 for various times and then observed by fluorescence microscopy. The proportion of cells with GFP-LC3 puncta is shown as a percentage of total GFP-expressing and GAS-infected HMEC-1 cells. Scale bar, 10 μm. (D) HMEC-1 cells were infected with GAS for 1 h, and then treated with gentamicin to kill extracellular bacteria. Cells were collected at the indicated time points post-infection and fixed for electron microscopy. White arrowheads indicate GAS within vesicles at early stages, and black arrows indicate GAS in the cytoplasm in late stage. No isolation membrane was detected at any time point post-infection. GAS division occurs at all stages post-infection. Scale bar, 5 μm for upper and 1 μm for below. (TIF) [file ppat.1006444.s002.tif]

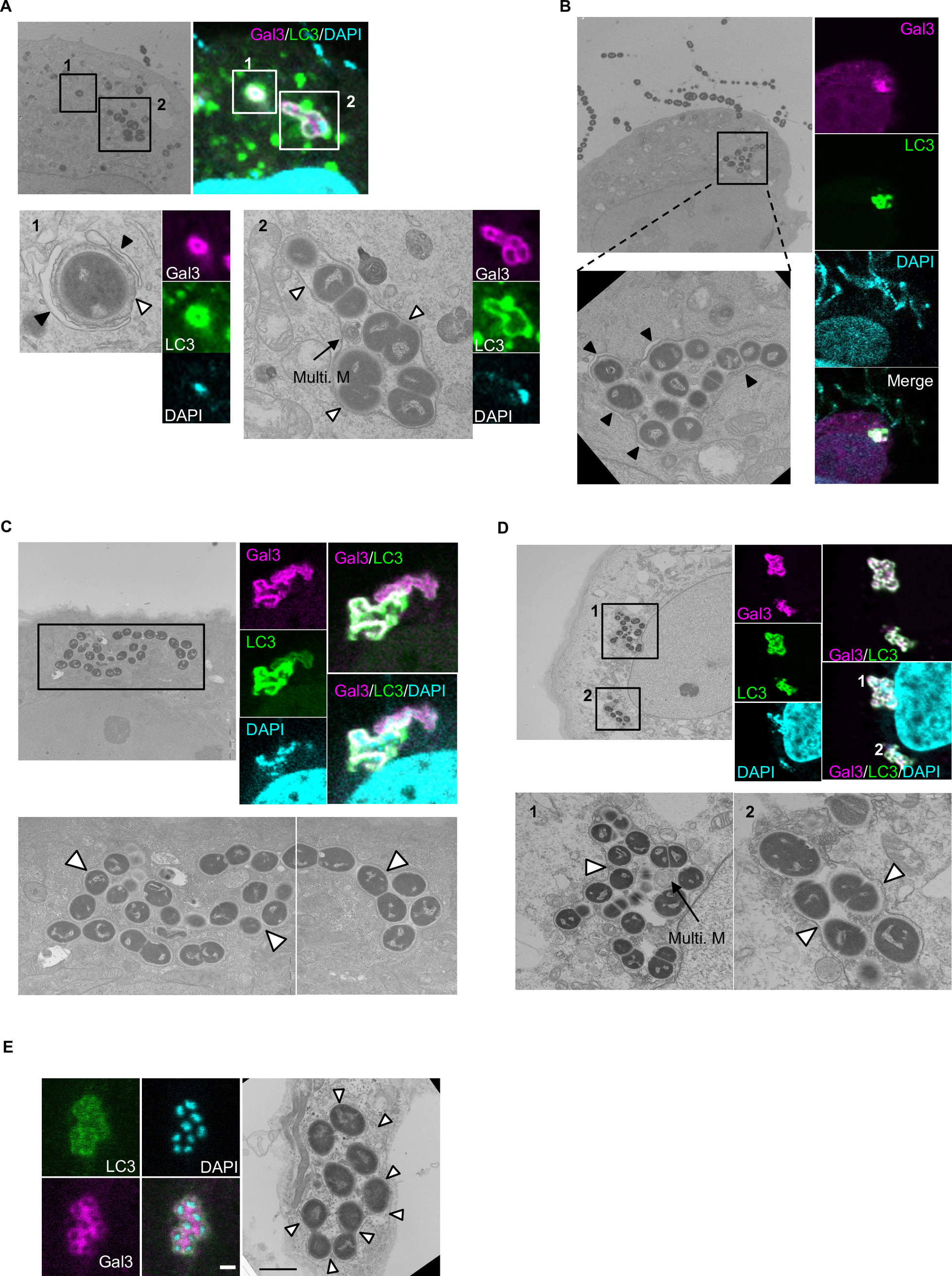

Supplement: S3 Fig — (A-D) Representative images of correlative light electron microscopy of GAS-infected cells. GFP-LC3 and Strawberry-Gal3 stably expressing A549 cells (A and B), HMEC-1 cells (C and D) and HUVEC cells (E) were cultured on gridded-glass bottom dishes, and then infected with GAS for 1 h. Cells were fixed and stained with DAPI for confocal microscopy. GFP-LC3 and Strawberry-Gal3 double-positive GAS were selected as targets for transmission electron microscopy. Black arrowheads indicate isolation membrane (double membrane structure), black arrows indicate multiple membrane structures inside the LC3/Gal3-decorated single membrane indicated by white arrowheads. (TIF) [file ppat.1006444.s003.tif]

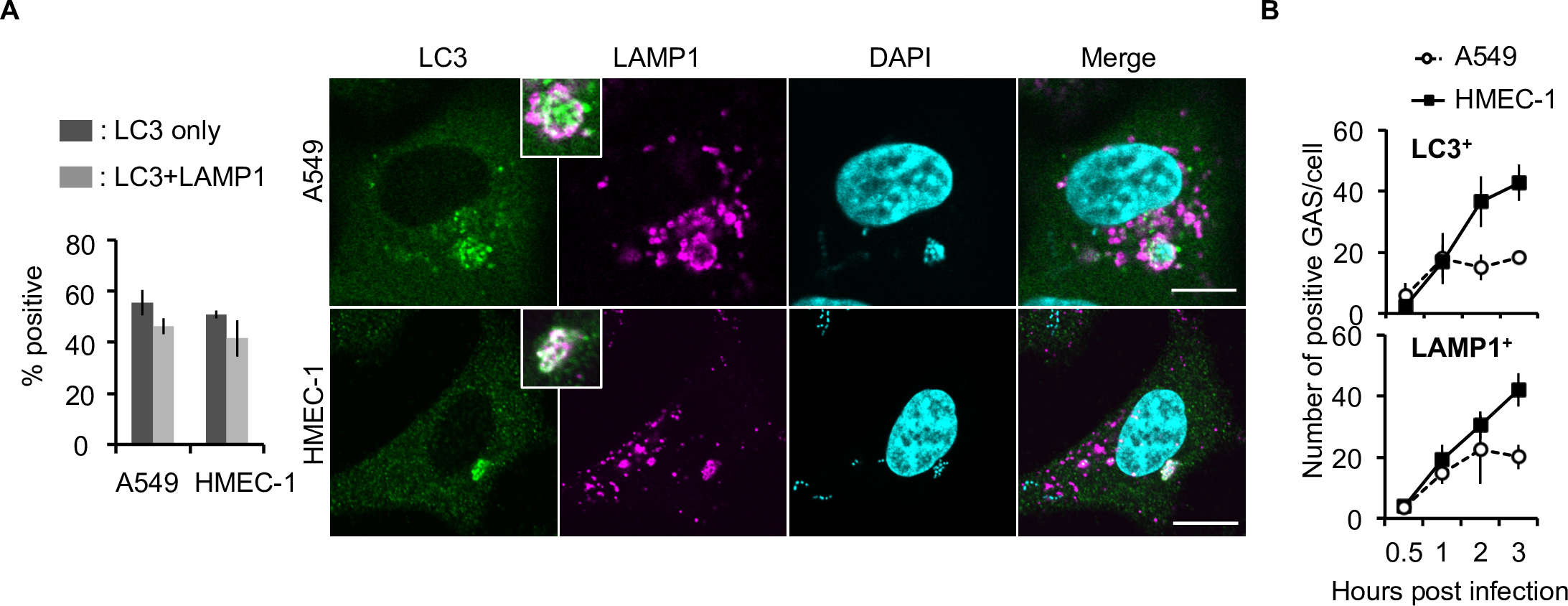

Supplement: S4 Fig — (A) The defect in GAS clearance in endothelial cells is correlated with accumulation of LC3- and LAMP1-positive GAS. Both A549 and HMEC-1 cells were positive for LC3 and LAMP1. At 1 h post-infection with GAS, cells were fixed and immunostained with anti-LC3 and anti-LAMP1 antibodies. Scale bar, 10 μm. (B) Intracellular GAS with LC3 (Top) or LAMP1 (bottom) were counted at the indicated time points post-infection. All quantitative data represent means ± SD from three independent experiments; more than 100 cells were counted in each sample. (TIF) [file ppat.1006444.s004.tif]

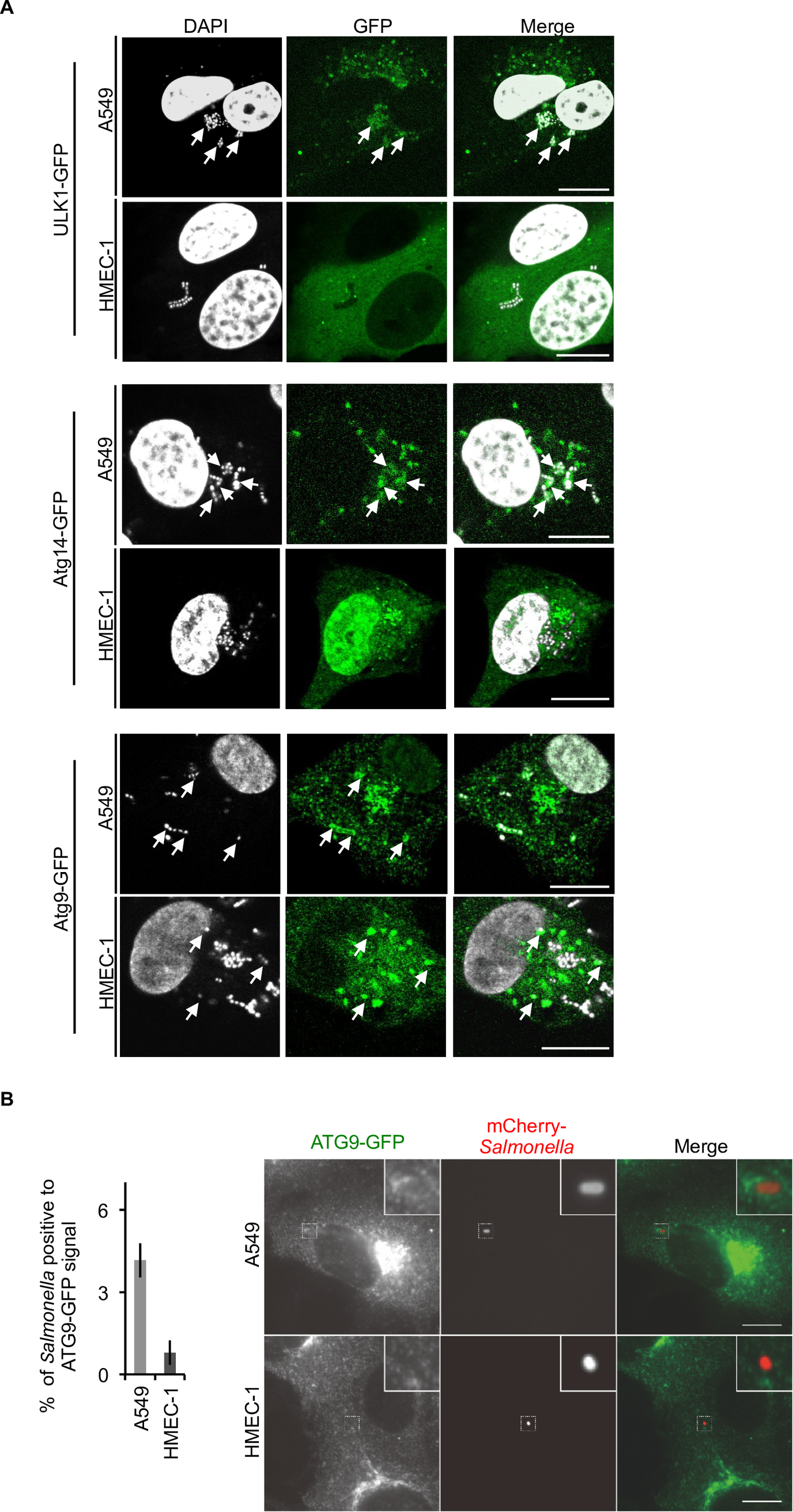

Supplement: S5 Fig — Cells with ectopic expression of indicated GFP-tagged proteins were infected with GAS (A) or Salmonella (B) for 1 h, and then examined for GFP signal on GAS within their cytoplasm. Images were acquired by confocal microscopy. Scale bars, 10 μm. Percentages of ATG9-GFP positive Salmonella were shown in (B). All quantitative data represent means ± SD from three independent experiments. (TIF) [file ppat.1006444.s005.tif]

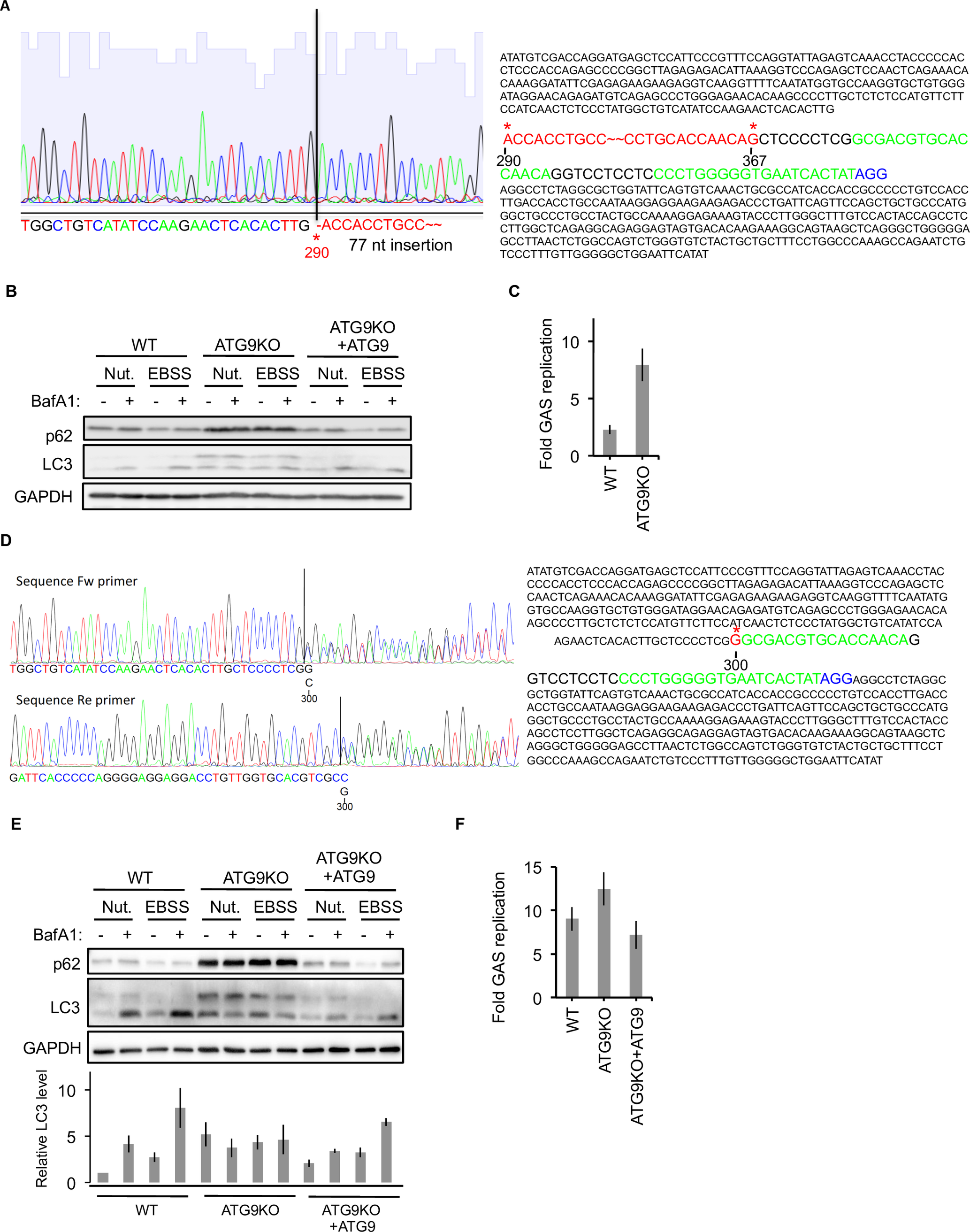

Supplement: S6 Fig — (A) Isolated HeLa-Kyoto cells harbor an insertion at the indicated locus in the first exon of ATG9. PAM sequence and recognition sequence are labeled in blue and green, respectively. (B) Autophagic flux was measured by p62 and LC3 degradation under nutrient-replete or starvation conditions. We observed no induction of LC3 II formation, and accumulation of p62, under starved conditions in ATG9-KO cells. This phenotype was rescued by ectopic expression of full-length ATG9. (C) HeLa-Kyoto ATG9-KO cells exhibited an increase GAS growth. Colony-forming assay (CFA) was performed at 1 and 6 h post-infection. Fold replication of GAS was calculated by comparison of GAS number at 6 h vs. 1 h. Error bars indicate SD from three independent experiments. (D) Isolated HMEC-1 cells contain one-nucleotide insertion at the indicated locus on the first exon of ATG9 gene. The PAM and recognition sequence are labeled in blue and green, respectively. (E) The ATG9-KO cell line exhibited no autophagic flux of p62 and a lack of LC3 lipidation. This phenotype was rescued by ectopic expression of full-length ATG9. (F) ATG9-KO HMEC-1 cells exhibited only a subtle increase in GAS growth relative to that in control wild-type cells. CFA was performed at 1 and 6 h post-infection. Fold replication of GAS was calculated by comparison of GAS number at 6 h vs. 1 h. Error bars indicate SD from three independent experiments. (TIF) [file ppat.1006444.s006.tif]

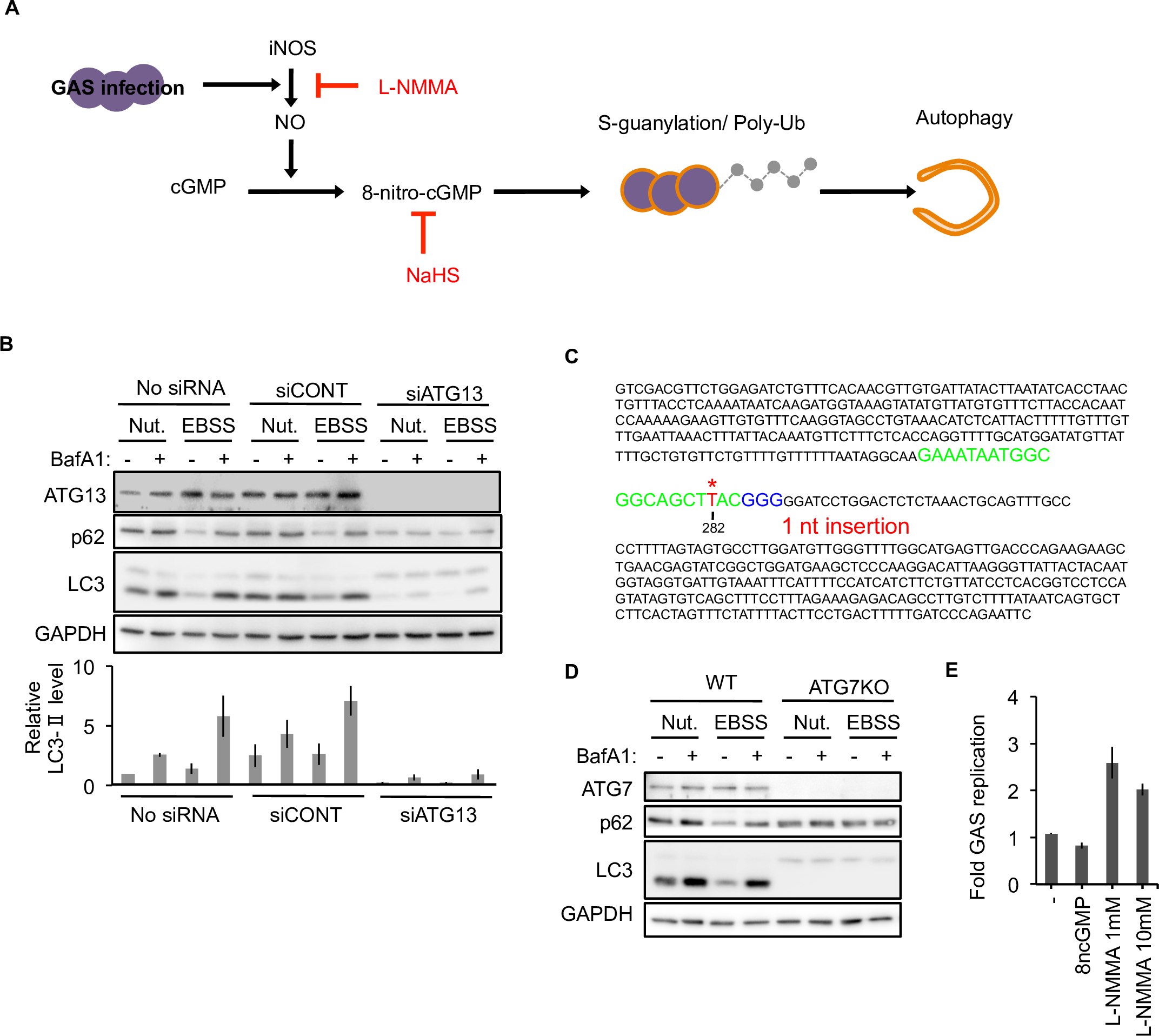

Supplement: S7 Fig — (A) GAS infection induces intracellular nitric oxide (NO), which is a short-lived reactive molecule that can readily be combined with cGMP to form 8-nitroguanosine 3′,5′-cyclic monophosphate (8-nitro-cGMP). Elevated levels of endogenous 8-nitro-cGMP can increase formation of LC3 puncta and autophagy under unstressed conditions. Furthermore, this endogenous nitrated nucleotide can also modify Cys residues on GAS surface molecules by S-guanylation, which promotes ubiquitination and contributes to bacterial clearance by xenophagy. L-NMMA inhibits suppression of nitric oxide synthase (NOS) activity. NaHS provides sulfhydryl anion HS- to degrade 8-nitro-cGMP into 8-SH-cGMP. (B) A549 cells were transfected with negative control siRNA or siRNA against Atg13 for 4 h, and then the medium was replaced prior to overnight culture. A secondary transfection was performed using the same protocol. Forty-eight hours after secondary transfection, cells were treated with complete DMEM medium or EBSS medium, with or without BafA1, for 2 h. Cell pellets were collected for determination of ATG13 (anti-ATG13 antibody, SAB4200, Sigma-Aldrich), p62, and LC3 protein levels by western blot assay. (C) When the CRISPR-Cas9 system was used to edit the first exon of the ATG7 gene, there was only one thymine insertion at nucleotide position 282 (red). PAM sequence and recognition sequence are labeled in blue and green, respectively. (D) Autophagic flux was detected by western blotting for p62 and LC3 II form under nutrient-replete or starvation conditions in cells treated or not treated with BafA1. The protein level of ATG7 was also confirmed by western blotting. No formation of LC3 II or change in p62 levels was observed in BafA1-treated ATG7-KO cells. (E) A high dose of L-NMMA is not necessary for inhibition of NOS in GAS-infected A549 cells. CFA was performed with or without drug treatments (8-nicro-cGMP, 100 μM; L-NMMA, 1 or 10 mM). Data represent means ± SD from three independent experiments. [file ppat.1006444.s007.tif]
